# 2-[1′-(Benz­yloxy)spiro­[indane-1,2′-pyrrolidine]-5′-yl]aceto­nitrile

**DOI:** 10.1107/S1600536813017674

**Published:** 2013-07-03

**Authors:** Rodolfo Moreno-Fuquen, Diana M. Soto, Luz M. Jaramillo-Gómez, Javier Ellena, Juan C. Tenorio

**Affiliations:** a Departamento de Química, Facultad de Ciencias, Universidad del Valle, Apartado 25360, Santiago de Cali, Colombia; b Instituto de Física de São Carlos, IFSC, Universidade de São Paulo, USP, São Carlos, SP, Brazil

## Abstract

In the title compound, C_21_H_22_N_2_O, the planes of the two six-membered rings make a dihedral angle of 89.51 (7)°. The pyrrolidine ring has a puckering amplitude *q*_2_ = 0.418 (3) and a pseudo-rotation phase angle ϕ_2_ = −166.8 (5), adopting a twist conformation (*T*). The other five-membered ring has a puckering amplitude *q*_2_ = 0.247 (2) and a pseudo-rotation phase angle ϕ_2_ = −173.7 (5), adopting an envelope conformation with the CH_2_ atom adjacent to the C atom common with the pyrrolidine ring as the flap. In the crystal, mol­ecules are linked *via* C—H⋯N, enclosing *R* ^2^_2_(20) rings, forming chains propagating along [100]. The aceto­nitrile group is disordered over two positions and was refined with a fixed occupancy ratio of 0.56:0.44.

## Related literature

For radical cyclization of 1-aza­spiro compounds, see: El Bialy *et al.* (2004 ▶); Dake (2006 ▶). For cephalotaxine synthesis, see: Paudler *et al.* (1963 ▶); Planas *et al.* (2004 ▶). For esters with anti­leukemic activity, see: Benderra *et al.* (1998 ▶); Kantarjian *et al.* (2001 ▶); Lévy *et al.* (2006 ▶). For pyrrolidine properties, see: Chen *et al.* (2012 ▶); Boyd *et al.* (1999 ▶). For tandem reactions under radical conditions, see: Jaramillo-Gómez *et al.* (2006 ▶). For bond-length data, see: Allen *et al.* (1987 ▶). For hydrogen bonding, see: Nardelli (1995 ▶) and for hydrogen-bond motifs, see: Etter (1990 ▶). For ring torsion angles, see: Cremer & Pople (1975 ▶).
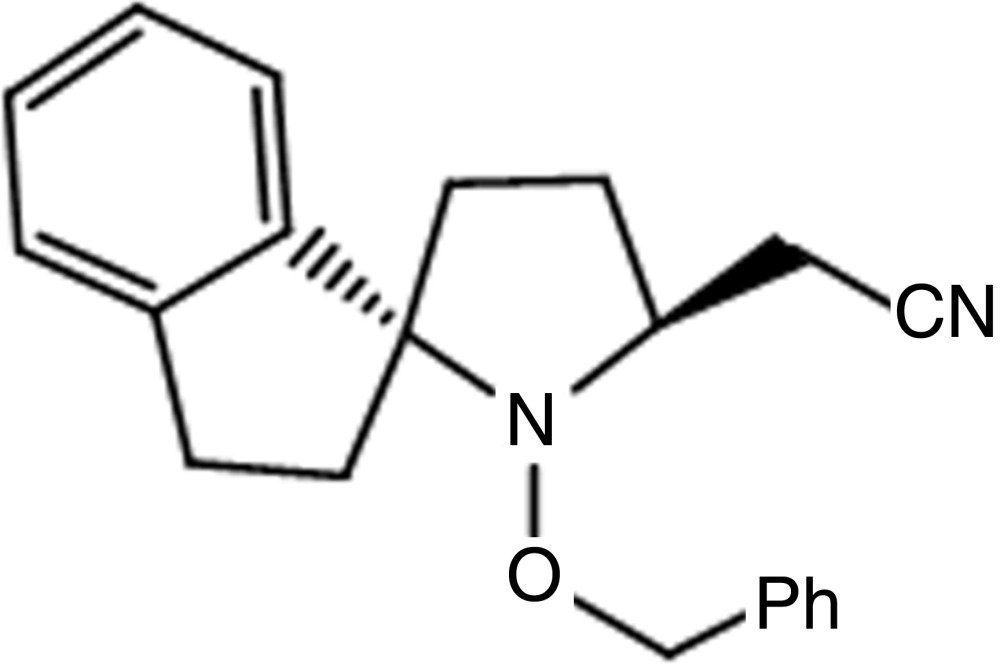


## Experimental

### 

#### Crystal data

C_21_H_22_N_2_O
*M* *_r_* = 318.41
Triclinic, 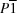

*a* = 9.1688 (4) Å
*b* = 10.0800 (4) Å
*c* = 11.4141 (6) Å
α = 98.826 (2)°
β = 108.777 (2)°
γ = 110.403 (4)°
*V* = 893.17 (7) Å^3^
*Z* = 2
Mo *K*α radiation
μ = 0.07 mm^−1^
*T* = 295 K
0.29 × 0.25 × 0.15 mm

#### Data collection

Nonius KappaCCD diffractometer
6429 measured reflections
3617 independent reflections
2307 reflections with *I* > 2σ(*I*)
*R*_int_ = 0.063

#### Refinement

*R*[*F* ^2^ > 2σ(*F* ^2^)] = 0.059
*wR*(*F* ^2^) = 0.191
*S* = 1.05
3617 reflections
249 parameters
3 restraints
H atoms treated by a mixture of independent and constrained refinement
Δρ_max_ = 0.11 e Å^−3^
Δρ_min_ = −0.13 e Å^−3^

### 

Data collection: *COLLECT* (Nonius, 2000 ▶); cell refinement: *SCALEPACK* (Otwinowski & Minor, 1997 ▶); data reduction: *DENZO* (Otwinowski & Minor, 1997 ▶) and *SCALEPACK*; program(s) used to solve structure: *SHELXS97* (Sheldrick, 2008 ▶); program(s) used to refine structure: *SHELXL97* (Sheldrick, 2008 ▶); molecular graphics: *ORTEP-3 for Windows* (Farrugia, 2012 ▶) and *Mercury* (Macrae *et al.*, 2006 ▶); software used to prepare material for publication: *WinGX* (Farrugia, 2012 ▶).

## Supplementary Material

Crystal structure: contains datablock(s) I, global. DOI: 10.1107/S1600536813017674/hg5325sup1.cif

Structure factors: contains datablock(s) I. DOI: 10.1107/S1600536813017674/hg5325Isup2.hkl

Supplementary material file. DOI: 10.1107/S1600536813017674/hg5325Isup3.cml

Additional supplementary materials:  crystallographic information; 3D view; checkCIF report

## Figures and Tables

**Table 1:** Hydrogen-bond geometry (Å, °)

*D*—H⋯*A*	*D*—H	H⋯*A*	*D*⋯*A*	*D*—H⋯*A*
C21—H21⋯N2*B* ^i^	0.93	2.61	3.511 (15)	164
C21—H21⋯N2*A* ^i^	0.93	2.48	3.390 (18)	168

Symmetry codes: (i) 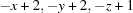.

**Fig. 1.**
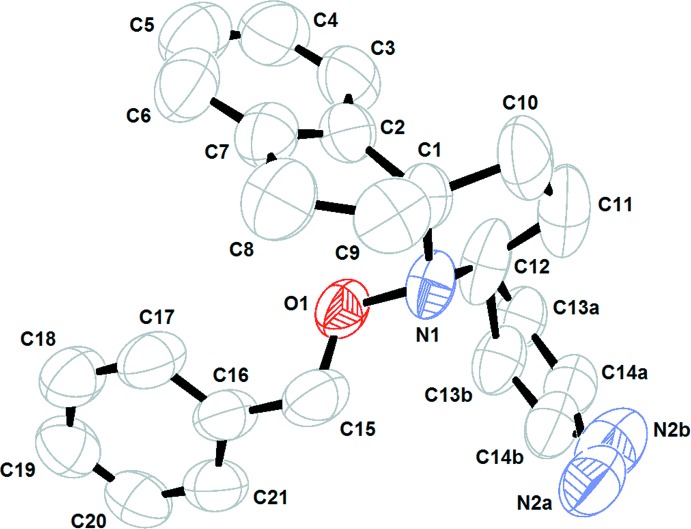
Molecular conformation and atom numbering scheme for the title compound with displacement ellipsoids drawn at the 50% probability level. H atoms were omitted for more clarity.

**Fig. 2.**
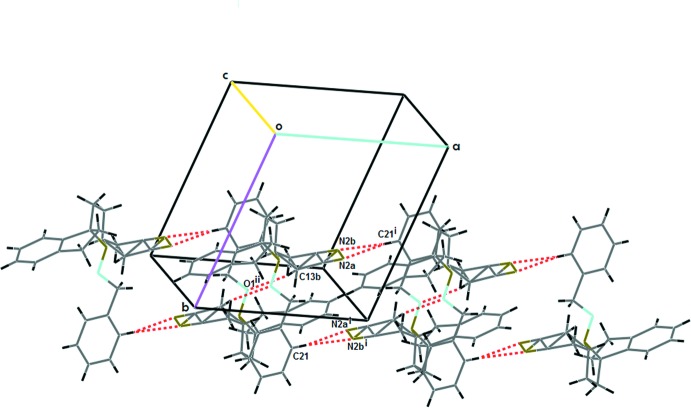
Part of the crystal structure of (IIIa), forming layers in the ab plane. Symmetry code: (i) -x+2,-y+2,-z+1; (ii) -x+1,-y+2,-z+1.

**Fig. 3.**
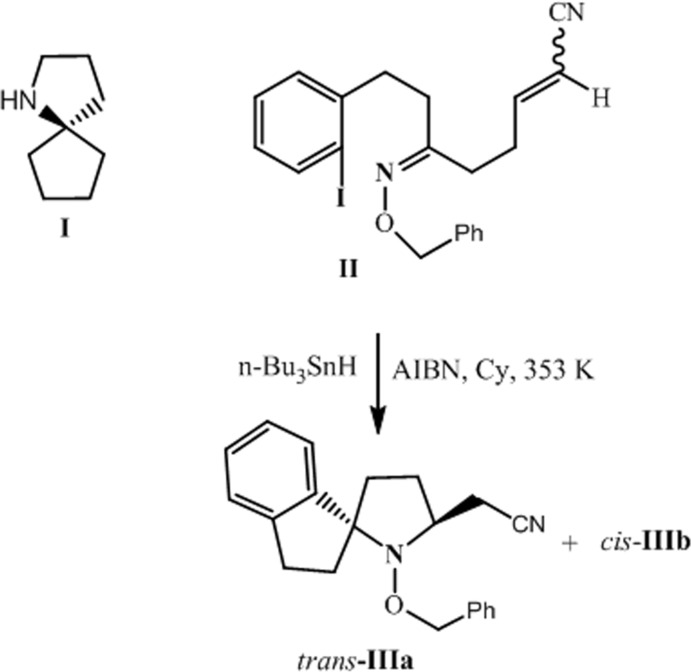
Reaction scheme.

## supplementary crystallographic information

### Comment

The title compound, 1'-(benzyloxy)-5'-cyanomethyl-2,3-dihydrospiro [inden-1,2'-pyrrolidine], (*trans-IIIa*), belongs to the family of spirocyclic compounds. It is worthwhile to stand out the natural occurring and synthetic cyclic alkaloids which contain a nitrogen atom adjacent to the spiro carbon. Of special interest are the structures exhibiting the 1-azaspiro[4.4]nonane (I) system (El Bialy *et al.*, 2004) which represents the central core of the cephalotaxine, a natural occurring product isolated from evergreen plum yews of the genus Cephalotaxus (Paudler *et al.*, 1963; Planas *et al.*, 2004), whose ester derivatives as homoharringtonine exhibited a pronounced antileukemic activity (Benderra *et al.*, 1998; Kantarjian *et al.*, 2001; Lévy *et al.*, 2006). Pyrrolidine is a heterocyclic amine used as building block or base in pharmaceutical and fine chemical manufacturing (Boyd *et al.*, 1999; Chen *et al.*, 2012). Therefore, considerable attention has been focused toward the synthesis of molecules with the embedded 1-azaspiro [4.4]nonane (I) system in their structures, using mainly ionic strategy and only scarce examples via radical cyclisation to form out them (El Bialy *et al.*, 2004; Dake, 2006).

Continuing with our current interest in applying tandem reactions under radical conditions, with the participation of aryl and neutral alkyl oxyaminyl radicals (Jaramillo-Gómez *et al.* 2006), we are reporting here, the synthesis of the azaspirocyclic 1'-(benzyloxy)-5'-cyanomethyl-2,3-dihydrospiro [inden-1,2'-pyrrolidine] (III), as a mixture of the diastereomers *cis* and *trans*, being able to crystallize in the pure form, the isomer *trans* IIIa. The interesting azaspiro [4.4] nonano (I) framework embedded in (III) was obtained in one single synthetic step, from the oxime ether 6-(benzyloxy-imino)-8-(2-iodophenyl)oct-2-enenitrile (II), through a sequential process of two closures 5-*exo* under standard radical conditions, (scheme 2). The molecular structure of (*trans* IIIa) is shown in Fig. 1.

The title compound crystallizes in the monoclinic space group P2_1_/c. The two phenyl rings are oriented to each other with a dihedral angle of 89.51 (7)°. Analysis of torsion angles, and least-square plane calculation, indicate that pyrrolidine ring shows a puckering amplitude q_2_= 0.418 (3) and pseudo-rotation phase angle φ_2_= -166.8 (5) adopting a twist conformation T with the N atom above the mean plane of the ring. In the same way the other five member ring shows a puckering amplitude q_2_= 0.247 (2) and pseudo-rotation phase angle φ_2_= -173.7 (5) adopting an envelope conformation E with the atom C9 below the mean plane of the ring (Cremer & Pople, 1975). The N-O bond length is close to the mean value [1.463 (12) Å] reported in the literature (Allen *et al.*, 1987) and the torsion angle formed by the atoms [N1-O1-C15-C16] which links the pyrrolidine and phenyl rings is 179.41 (13)°. The crystal packing reveals that the molecules are linked through a network of weak C—H···N and C—H···O intermolecular interactions (see Table 1, Nardelli, 1995). The C21 atom in the molecule at (x,y,z) donates a proton to N2a y N2b atoms in the molecule at (-x+2,-y+2,-z+1), forming as a result of these interactions R^2^_2_(20) rings (Etter, 1990). These rings are in turn linked by a weak C-H···O interaction. Indeed, the C23 atom in the molecule at (x,y,z) donates a proton to O1 atom of the molecule at (-x+1,-y+2,-z+1) forming layers parallel to (001) as shown in Fig. 2.

### Experimental

The reagents and solvents for the synthesis were obtained from the Aldrich Chemical Co., and were used without additional purification. A solution of 6-(benzyloxyimino)-8-(2-iodophenyl)oct-2-enenitrile (II) (129 mg, 0.29 mmol), 2,2'-azobisisobutyronitrile (AIBN, 14 mg, 0.09 mmol) and tributyltin hydride (n-Bu_3_SnH, 0.09 ml, 0.35 mmol) in cyclohexane (Cy, 13 mL) was degassed for 1 h by bubbling dry argon, and subsequently stirred at 353 K for 7 h. After cooling to room temperature the solvent was removed under reduced pressure and the crude product treated with a mixture of 20% KF aqueous solution (2 mL) and ethyl acetate (2 mL), stirring overnight. The organic layer was separated, dried with anhydrous Na_2_SO_4_ and filtered over silica gel. The purification was carried out by flash column chromatography with 60-95% benzene/hexane (gradient 5%) to afford a mixture of two diastereomers IIIa and IIIb (59 mg, 65%) as yellow oil. By addition of hexane to this oil, white crystals suitable for X-ray diffraction, fell down [20 mg, 21%, m.p. 372 (1) K] of the diastereomeric spirocycle *trans*-IIIa suitable for X-ray analysis. Elemental Analysis: Calculated: C 79.20, H 6.98, N 8.80; Found: C 79.25, H 6.73, N 8.85.

### Refinement

The H-atoms were positioned geometrically [C—H= 0.93 Å for aromatic and C—H= 0.97 Å for methylene, and with U_iso_(H) (1.2 and 1.5 times U_eq_ of the parent atom respectively]. The H12 atom was found in difference Fourier maps an its coordinates were refined freely.

### Crystal data

**Table:**

C_21_H_22_N_2_O	*Z* = 2
*M**_r_* = 318.41	*F*(000) = 340
Triclinic, *P*1	*D*_x_ = 1.184 Mg m^−^^3^
Hall symbol: -P 1	Melting point: 372(1) K
*a* = 9.1688 (4) Å	Mo *K*α radiation, λ = 0.71073 Å
*b* = 10.0800 (4) Å	Cell parameters from 5157 reflections
*c* = 11.4141 (6) Å	θ = 2.9–25.7°
α = 98.826 (2)°	µ = 0.07 mm^−^^1^
β = 108.777 (2)°	*T* = 295 K
γ = 110.403 (4)°	Block, white
*V* = 893.17 (7) Å^3^	0.29 × 0.25 × 0.15 mm

### Data collection

**Table:**

Nonius KappaCCD diffractometer	2307 reflections with *I* > 2σ(*I*)
Radiation source: fine-focus sealed tube	*R*_int_ = 0.063
Graphite monochromator	θ_max_ = 26.4°, θ_min_ = 3.0°
CCD rotation images, thick slices scans	*h* = −11→10
6429 measured reflections	*k* = −12→12
3617 independent reflections	*l* = −14→14

### Refinement

**Table:**

Refinement on *F*^2^	Primary atom site location: structure-invariant direct methods
Least-squares matrix: full	Secondary atom site location: difference Fourier map
*R*[*F*^2^ > 2σ(*F*^2^)] = 0.059	Hydrogen site location: inferred from neighbouring sites
*wR*(*F*^2^) = 0.191	H atoms treated by a mixture of independent and constrained refinement
*S* = 1.05	*w* = 1/[σ^2^(*F*_o_^2^) + (0.0894*P*)^2^ + 0.0489*P*] where *P* = (*F*_o_^2^ + 2*F*_c_^2^)/3
3617 reflections	(Δ/σ)_max_ < 0.001
249 parameters	Δρ_max_ = 0.11 e Å^−^^3^
3 restraints	Δρ_min_ = −0.13 e Å^−^^3^

### Special details

**Table:**

Geometry. All esds (except the esd in the dihedral angle between two l.s. planes) are estimated using the full covariance matrix. The cell esds are taken into account individually in the estimation of esds in distances, angles and torsion angles; correlations between esds in cell parameters are only used when they are defined by crystal symmetry. An approximate (isotropic) treatment of cell esds is used for estimating esds involving l.s. planes.
Refinement. Refinement of *F*^2^ against ALL reflections. The weighted *R*-factor *wR* and goodness of fit *S* are based on *F*^2^, conventional *R*-factors *R* are based on *F*, with *F* set to zero for negative *F*^2^. The threshold expression of *F*^2^ > σ(*F*^2^) is used only for calculating *R*-factors(gt) *etc*. and is not relevant to the choice of reflections for refinement. *R*-factors based on *F*^2^ are statistically about twice as large as those based on *F*, and *R*- factors based on ALL data will be even larger.

### Fractional atomic coordinates and isotropic or equivalent isotropic displacement parameters (Å^2^)

**Table:**

	*x*	*y*	*z*	*U*_iso_*/*U*_eq_	Occ. (<1)
O1	0.48268 (14)	0.93003 (12)	0.30050 (11)	0.0770 (4)	
N1	0.46487 (19)	0.78319 (16)	0.30577 (14)	0.0779 (4)	
C1	0.2926 (2)	0.66924 (19)	0.20916 (18)	0.0789 (5)	
C2	0.1560 (2)	0.72661 (18)	0.18692 (19)	0.0784 (5)	
C3	0.0707 (3)	0.7469 (2)	0.2642 (2)	0.1021 (7)	
H3	0.0910	0.7198	0.3401	0.123*	
C4	−0.0441 (3)	0.8075 (3)	0.2276 (3)	0.1190 (8)	
H4	−0.1011	0.8212	0.2794	0.143*	
C5	−0.0749 (3)	0.8472 (3)	0.1174 (3)	0.1224 (9)	
H5	−0.1503	0.8905	0.0955	0.147*	
C6	0.0039 (3)	0.8241 (3)	0.0373 (2)	0.1114 (8)	
H6	−0.0198	0.8491	−0.0396	0.134*	
C7	0.1202 (2)	0.7625 (2)	0.07324 (19)	0.0878 (5)	
C8	0.2196 (3)	0.7262 (3)	0.0030 (2)	0.1071 (7)	
H8A	0.3110	0.8155	0.0083	0.128*	
H8B	0.1470	0.6717	−0.0876	0.128*	
C9	0.2901 (3)	0.6305 (2)	0.07358 (19)	0.0958 (6)	
H9A	0.2181	0.5260	0.0281	0.115*	
H9B	0.4041	0.6519	0.0789	0.115*	
C10	0.2684 (4)	0.5431 (3)	0.2722 (3)	0.1171 (8)	
H10A	0.1538	0.5014	0.2686	0.141*	
H10B	0.2867	0.4648	0.2269	0.141*	
C11	0.3975 (4)	0.6097 (3)	0.4117 (3)	0.1284 (10)	
H11A	0.3433	0.5835	0.4703	0.154*	
H11B	0.4862	0.5751	0.4251	0.154*	
C12	0.4700 (4)	0.7747 (3)	0.4335 (2)	0.1040 (7)	
C15	0.6189 (2)	0.9965 (2)	0.2623 (2)	0.0990 (7)	
H15A	0.5962	0.9367	0.1778	0.119*	
H15B	0.7253	1.0050	0.3246	0.119*	
C16	0.6290 (2)	1.1473 (2)	0.2575 (2)	0.0853 (5)	
C17	0.4913 (2)	1.1636 (2)	0.1765 (2)	0.1015 (7)	
H17	0.3909	1.0799	0.1244	0.122*	
C18	0.5008 (3)	1.3020 (2)	0.1720 (2)	0.1035 (7)	
H18	0.4072	1.3108	0.1167	0.124*	
C19	0.6473 (3)	1.4269 (2)	0.2482 (2)	0.0987 (6)	
H19	0.6535	1.5203	0.2450	0.118*	
C20	0.7837 (3)	1.4125 (2)	0.3289 (2)	0.0985 (7)	
H20	0.8833	1.4968	0.3811	0.118*	
C21	0.7759 (2)	1.2749 (2)	0.3340 (2)	0.0913 (6)	
H21	0.8704	1.2674	0.3896	0.110*	
N2A	0.8984 (16)	0.801 (2)	0.4992 (18)	0.115 (3)	0.44
C13A	0.6184 (11)	0.8347 (10)	0.5469 (9)	0.097 (2)	0.44
H131	0.5934	0.8012	0.6163	0.116*	0.44
H132	0.6644	0.9421	0.5727	0.116*	0.44
C14A	0.7383 (11)	0.7889 (10)	0.5243 (10)	0.088 (2)	0.44
N2B	0.8463 (15)	0.7591 (16)	0.5103 (16)	0.131 (3)	0.56
C13B	0.6718 (10)	0.8878 (9)	0.5325 (7)	0.1077 (18)	0.56
H133	0.6979	0.9843	0.5184	0.129*	0.56
H134	0.6827	0.9002	0.6214	0.129*	0.56
C14B	0.7995 (10)	0.8369 (8)	0.5167 (8)	0.0904 (18)	0.56
H12	0.400 (3)	0.821 (2)	0.454 (2)	0.109 (6)*	

### Atomic displacement parameters (Å^2^)

**Table:**

	*U*^11^	*U*^22^	*U*^33^	*U*^12^	*U*^13^	*U*^23^
O1	0.0734 (7)	0.0694 (7)	0.0904 (8)	0.0416 (6)	0.0252 (6)	0.0203 (6)
N1	0.0945 (10)	0.0754 (9)	0.0749 (9)	0.0552 (8)	0.0267 (8)	0.0232 (7)
C1	0.0921 (12)	0.0670 (10)	0.0862 (12)	0.0423 (9)	0.0369 (10)	0.0232 (8)
C2	0.0791 (10)	0.0624 (9)	0.0939 (13)	0.0315 (8)	0.0337 (9)	0.0232 (8)
C3	0.1121 (15)	0.0891 (13)	0.1370 (18)	0.0530 (12)	0.0723 (14)	0.0453 (13)
C4	0.1025 (16)	0.1018 (17)	0.175 (3)	0.0558 (14)	0.0705 (17)	0.0389 (17)
C5	0.0930 (15)	0.1002 (16)	0.161 (3)	0.0525 (13)	0.0300 (16)	0.0244 (17)
C6	0.1009 (15)	0.0993 (16)	0.1069 (16)	0.0462 (13)	0.0078 (13)	0.0261 (13)
C7	0.0812 (11)	0.0782 (11)	0.0837 (12)	0.0311 (9)	0.0157 (9)	0.0162 (9)
C8	0.1162 (15)	0.1236 (18)	0.0698 (12)	0.0529 (13)	0.0265 (10)	0.0182 (11)
C9	0.1021 (13)	0.0907 (13)	0.0840 (13)	0.0428 (11)	0.0335 (11)	0.0031 (10)
C10	0.157 (2)	0.0902 (15)	0.156 (2)	0.0813 (15)	0.084 (2)	0.0627 (15)
C11	0.203 (3)	0.153 (2)	0.135 (2)	0.135 (2)	0.107 (2)	0.0954 (19)
C12	0.1553 (19)	0.1313 (18)	0.0756 (12)	0.1116 (16)	0.0472 (12)	0.0407 (11)
C15	0.0703 (11)	0.0884 (14)	0.1379 (18)	0.0410 (10)	0.0376 (11)	0.0254 (12)
C16	0.0615 (9)	0.0775 (11)	0.1134 (15)	0.0298 (8)	0.0346 (9)	0.0204 (10)
C17	0.0653 (10)	0.0742 (12)	0.1411 (18)	0.0269 (9)	0.0232 (11)	0.0170 (11)
C18	0.0837 (12)	0.0872 (14)	0.1346 (18)	0.0407 (11)	0.0354 (12)	0.0290 (12)
C19	0.1060 (15)	0.0746 (12)	0.1174 (16)	0.0322 (11)	0.0545 (14)	0.0286 (11)
C20	0.0870 (13)	0.0801 (13)	0.1029 (15)	0.0109 (10)	0.0380 (12)	0.0208 (11)
C21	0.0659 (10)	0.0980 (14)	0.0982 (14)	0.0245 (10)	0.0316 (10)	0.0269 (11)
N2A	0.096 (7)	0.119 (8)	0.122 (5)	0.060 (6)	0.022 (5)	0.029 (5)
C13A	0.146 (5)	0.111 (6)	0.080 (3)	0.097 (4)	0.048 (2)	0.041 (3)
C14A	0.086 (5)	0.081 (5)	0.090 (4)	0.042 (4)	0.020 (4)	0.024 (4)
N2B	0.109 (7)	0.124 (8)	0.146 (6)	0.070 (6)	0.026 (5)	0.011 (5)
C13B	0.171 (3)	0.120 (5)	0.060 (2)	0.103 (3)	0.032 (3)	0.031 (3)
C14B	0.090 (5)	0.080 (4)	0.089 (3)	0.046 (3)	0.013 (3)	0.017 (3)

### Geometric parameters (Å, º)

**Table:**

O1—C15	1.434 (2)	C11—H11A	0.9700
O1—N1	1.4444 (17)	C11—H11B	0.9700
N1—C12	1.460 (3)	C12—C13A	1.395 (9)
N1—C1	1.502 (2)	C12—C13B	1.670 (8)
C1—C2	1.520 (2)	C12—H12	0.97 (2)
C1—C9	1.528 (3)	C15—C16	1.501 (3)
C1—C10	1.540 (3)	C15—H15A	0.9700
C2—C7	1.369 (3)	C15—H15B	0.9700
C2—C3	1.390 (3)	C16—C17	1.384 (3)
C3—C4	1.378 (3)	C16—C21	1.387 (3)
C3—H3	0.9300	C17—C18	1.377 (3)
C4—C5	1.351 (4)	C17—H17	0.9300
C4—H4	0.9300	C18—C19	1.371 (3)
C5—C6	1.375 (4)	C18—H18	0.9300
C5—H5	0.9300	C19—C20	1.364 (3)
C6—C7	1.396 (3)	C19—H19	0.9300
C6—H6	0.9300	C20—C21	1.374 (3)
C7—C8	1.491 (3)	C20—H20	0.9300
C8—C9	1.529 (3)	C21—H21	0.9300
C8—H8A	0.9700	N2A—C14A	1.553 (17)
C8—H8B	0.9700	C13A—C14A	1.410 (16)
C9—H9A	0.9700	C13A—H131	0.9700
C9—H9B	0.9700	C13A—H132	0.9700
C10—C11	1.514 (4)	N2B—C14B	1.021 (17)
C10—H10A	0.9700	C13B—C14B	1.481 (13)
C10—H10B	0.9700	C13B—H133	0.9700
C11—C12	1.505 (4)	C13B—H134	0.9700
			
C15—O1—N1	109.50 (12)	C10—C11—H11B	110.7
O1—N1—C12	107.46 (13)	H11A—C11—H11B	108.8
O1—N1—C1	110.06 (12)	C13A—C12—N1	124.5 (4)
C12—N1—C1	106.19 (16)	C13A—C12—C11	105.6 (4)
N1—C1—C2	112.94 (14)	N1—C12—C11	101.52 (17)
N1—C1—C9	111.30 (15)	N1—C12—C13B	103.0 (3)
C2—C1—C9	101.77 (16)	C11—C12—C13B	122.4 (3)
N1—C1—C10	100.66 (17)	C13A—C12—H12	103.9 (13)
C2—C1—C10	115.07 (17)	N1—C12—H12	107.9 (12)
C9—C1—C10	115.58 (17)	C11—C12—H12	113.9 (13)
C7—C2—C3	119.32 (19)	C13B—C12—H12	106.6 (13)
C7—C2—C1	111.24 (17)	O1—C15—C16	106.64 (14)
C3—C2—C1	129.44 (18)	O1—C15—H15A	110.4
C4—C3—C2	119.6 (2)	C16—C15—H15A	110.4
C4—C3—H3	120.2	O1—C15—H15B	110.4
C2—C3—H3	120.2	C16—C15—H15B	110.4
C5—C4—C3	120.8 (3)	H15A—C15—H15B	108.6
C5—C4—H4	119.6	C17—C16—C21	117.70 (19)
C3—C4—H4	119.6	C17—C16—C15	121.00 (17)
C4—C5—C6	120.7 (2)	C21—C16—C15	121.30 (18)
C4—C5—H5	119.6	C18—C17—C16	120.93 (19)
C6—C5—H5	119.6	C18—C17—H17	119.5
C5—C6—C7	118.9 (2)	C16—C17—H17	119.5
C5—C6—H6	120.5	C19—C18—C17	120.5 (2)
C7—C6—H6	120.5	C19—C18—H18	119.7
C2—C7—C6	120.5 (2)	C17—C18—H18	119.7
C2—C7—C8	110.62 (18)	C20—C19—C18	119.1 (2)
C6—C7—C8	128.8 (2)	C20—C19—H19	120.4
C7—C8—C9	103.76 (17)	C18—C19—H19	120.4
C7—C8—H8A	111.0	C19—C20—C21	120.88 (19)
C9—C8—H8A	111.0	C19—C20—H20	119.6
C7—C8—H8B	111.0	C21—C20—H20	119.6
C9—C8—H8B	111.0	C20—C21—C16	120.8 (2)
H8A—C8—H8B	109.0	C20—C21—H21	119.6
C1—C9—C8	106.36 (16)	C16—C21—H21	119.6
C1—C9—H9A	110.5	C12—C13A—C14A	109.0 (8)
C8—C9—H9A	110.5	C12—C13A—H131	109.9
C1—C9—H9B	110.5	C14A—C13A—H131	109.9
C8—C9—H9B	110.5	C12—C13A—H132	109.9
H9A—C9—H9B	108.6	C14A—C13A—H132	109.9
C11—C10—C1	106.99 (19)	H131—C13A—H132	108.3
C11—C10—H10A	110.3	C13A—C14A—N2A	158.1 (9)
C1—C10—H10A	110.3	C14B—C13B—C12	115.0 (6)
C11—C10—H10B	110.3	C14B—C13B—H133	108.5
C1—C10—H10B	110.3	C12—C13B—H133	108.5
H10A—C10—H10B	108.6	C14B—C13B—H134	108.5
C12—C11—C10	105.04 (17)	C12—C13B—H134	108.5
C12—C11—H11A	110.7	H133—C13B—H134	107.5
C10—C11—H11A	110.7	N2B—C14B—C13B	152.4 (10)
C12—C11—H11B	110.7		
			
C15—O1—N1—C12	127.02 (18)	C2—C1—C10—C11	−105.6 (2)
C15—O1—N1—C1	−117.76 (16)	C9—C1—C10—C11	136.2 (2)
O1—N1—C1—C2	−31.15 (19)	C1—C10—C11—C12	10.2 (3)
C12—N1—C1—C2	84.87 (17)	O1—N1—C12—C13A	−78.5 (5)
O1—N1—C1—C9	82.60 (17)	C1—N1—C12—C13A	163.7 (5)
C12—N1—C1—C9	−161.38 (16)	O1—N1—C12—C11	163.27 (17)
O1—N1—C1—C10	−154.38 (14)	C1—N1—C12—C11	45.5 (2)
C12—N1—C1—C10	−38.36 (19)	O1—N1—C12—C13B	−69.3 (3)
N1—C1—C2—C7	102.02 (18)	C1—N1—C12—C13B	173.0 (3)
C9—C1—C2—C7	−17.40 (19)	C10—C11—C12—C13A	−164.3 (5)
C10—C1—C2—C7	−143.16 (19)	C10—C11—C12—N1	−33.3 (2)
N1—C1—C2—C3	−77.2 (2)	C10—C11—C12—C13B	−146.9 (4)
C9—C1—C2—C3	163.37 (19)	N1—O1—C15—C16	179.41 (14)
C10—C1—C2—C3	37.6 (3)	O1—C15—C16—C17	−59.3 (3)
C7—C2—C3—C4	−2.2 (3)	O1—C15—C16—C21	120.56 (19)
C1—C2—C3—C4	176.98 (19)	C21—C16—C17—C18	0.3 (3)
C2—C3—C4—C5	0.1 (4)	C15—C16—C17—C18	−179.8 (2)
C3—C4—C5—C6	1.9 (4)	C16—C17—C18—C19	−0.3 (4)
C4—C5—C6—C7	−1.7 (4)	C17—C18—C19—C20	0.0 (4)
C3—C2—C7—C6	2.4 (3)	C18—C19—C20—C21	0.2 (3)
C1—C2—C7—C6	−176.96 (17)	C19—C20—C21—C16	−0.1 (3)
C3—C2—C7—C8	−177.52 (18)	C17—C16—C21—C20	−0.2 (3)
C1—C2—C7—C8	3.2 (2)	C15—C16—C21—C20	−179.98 (18)
C5—C6—C7—C2	−0.4 (3)	N1—C12—C13A—C14A	−49.7 (7)
C5—C6—C7—C8	179.4 (2)	C11—C12—C13A—C14A	66.6 (6)
C2—C7—C8—C9	12.6 (2)	C13B—C12—C13A—C14A	−73.4 (17)
C6—C7—C8—C9	−167.3 (2)	C12—C13A—C14A—N2A	105 (3)
N1—C1—C9—C8	−96.19 (19)	C13A—C12—C13B—C14B	93.7 (19)
C2—C1—C9—C8	24.4 (2)	N1—C12—C13B—C14B	−66.5 (5)
C10—C1—C9—C8	149.8 (2)	C11—C12—C13B—C14B	46.4 (6)
C7—C8—C9—C1	−23.2 (2)	C12—C13B—C14B—N2B	−51 (3)
N1—C1—C10—C11	16.2 (2)		

### Hydrogen-bond geometry (Å, º)

**Table:**

*D*—H···*A*	*D*—H	H···*A*	*D*···*A*	*D*—H···*A*
C21—H21···N2*B*^i^	0.93	2.61	3.511 (15)	164
C21—H21···N2*A*^i^	0.93	2.48	3.390 (18)	168
C13*B*—H134···O1^ii^	0.97	2.87	3.389 (9)	115

Symmetry codes: (i) −*x*+2, −*y*+2, −*z*+1; (ii) −*x*+1, −*y*+2, −*z*+1.
